# A HPLC-DAD method for the simultaneous determination of five marker components in the traditional herbal medicine Bangpungtongsung-san

**DOI:** 10.4103/0973-1296.75903

**Published:** 2011

**Authors:** Jin Bae Weon, Hye Jin Yang, Jin Yeul Ma, Choong Je Ma

**Affiliations:** 1 *Department of Biomaterials Engineering, Division of Biotechnology and Bioengineering, Kangwon National University, Chuncheon 200-701, South Korea*; 2 *Department of Biomaterials Engineering, Division of Biotechnology and Bioengineering, Kangwon National University, Chuncheon 200-701, South Korea*; 3 *TKM Converging Research Division, Korea Institute of Oriental Medicine, Daejeon 305-811, South Korea*; *Department of Biomaterials Engineering, Division of Biotechnology and Bioengineering; Research Institute of Bioscience and Biotechnology, Kangwon National University, Chuncheon 200-701, South Korea*

**Keywords:** Bangpungtongsung-san, diode-array detector, high-performance liquid chromatography, marker component, validation

## Abstract

**Background:**

Bangpungtongsung-san, one of the traditional herbal medicines, was known to be a prescription for obesity.

**Objective:**

For the simultaneous determination of five components (paeoniflorin, 6-gingerol, decursin, geniposide, and glycyrrhizin) in Bangpungtongsung-san, a high-performance liquid chromatography with diode-array detector method was established.

**Materials and Methods:**

To develop the method, a reverse phase column, DIONEX C _18_ (5 μm, 120 µ, 4.6 mm × 150 mm) was used. The mobile phase consisted of methanol and water using a gradient elution. The UV wavelength was set at 230, 240, and 254 nm. Method validation was accomplished by linearity, precision test, and recovery test.

**Results:**

All calibration curves of components showed good linearity (R ^2^ > 0.9959). The limit of detection (LOD) and limit of quantification (LOQ) ranged from 0.01 to 0.17 μg/ml and 0.04 to 0.53 μg/ml, respectively. The relative standard deviations (RSD) value of precision test, intraday and interday tests were less than 0.43% and 1.26%. In the recovery test, results of accuracy ranged from 95.27% to 107.70% with RSD values less than 2.21%.

**Conclusion:**

This developed method was applied to the commercial Bangpungtongsung-san sample and the five marker components were separated effectively without interference of any peaks of components.

## INTRODUCTION

**Figure 1:**
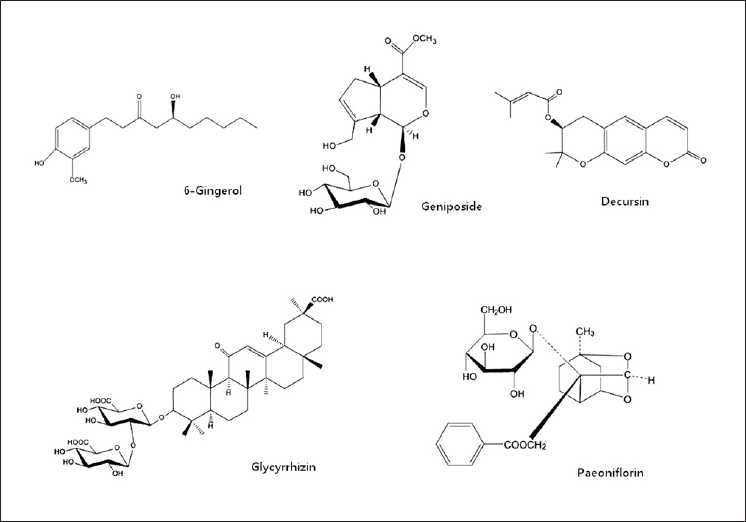
Chemical structures of five marker constituents in Bangpuntongsung-san

Bangpungtongsung-san was one of the major traditional herbal medicines wildly used in the treatment of obese patients.[1] Besides, it was used for the treatment of various diseases such as allergic rhinitis, hypertension, hyperlipidemia, atopic dermatitis, and used as an anticonvulsant, sedative, and analgesic.[2–4] According to the Korean Pharmacopoeia, Bangpungtongsung-san consisted of *Angelica gigas, *Paeonia lactiflora*, Cnidium officinale, Gardenia jasminoides, Forsythia viridissima, Mentha arvensis, Zingiber officinale, Schizonepeta tenuifolia, Saposhnikovia divaricata, Ephedra sinica, Rheum undulatum, Atractylodes japonica, Playtcodon grandiflorum, Scutellaria baicalensis, Glycyrrhiza uralensis, Gypsum, Talcum, and Natrii sulfas*.[5] Bangpungtongsung-san, the traditional herbal medicine, usually composed of multiple herbs. Therefore, the traditional herbal medicine cured many diseases by one formula, and had variety of formulas for one disease.[6] Nowadays, the commercial use of the traditional herbal medicine has increased and new various drugs are being developed from it; consequently, quality improvement of the traditional herbal medicine was required. For quality control, the chemical components in traditional herbal medicine were assayed. Because the traditional herbal medicine’s composition was very complicatedly and consisted of various chemical components, such as alkaloids, phenols, terpenoids, and flavonoids, we need to develop an accurate and effective simultaneous separation assay.[7] Many assay methods of traditional herbal medicine were researched lately.[8,10] In this study, a simultaneous determination method of five marker components, paeoniflorin from *Paeonia lactiflora*, 6-gingerol from *Zingiber officinale*, decursin from *Angelica gigas*, geniposide from *Gardenia jasminoides*, and glycyrrhizin from *Glycyrrhiza uralensis* in Banpungtongsung-san was established. The structures of these components are shown in Figure 1. To develop the assay method, we used high-performance liquid chromatography (HPLC) with a diode-array detector (DAD). Also we executed a validation of this HPLC-DAD method. The established method was used with Bangpungtodngsung-san samples.

## EXPERIMENT

### Reagents and standards

Four reference standards, paeoniflorin, glycyrrhizin, decursin, and 6-gingerol were purchased from Natural Product Chemistry BioTech Inc. (Seoul, South Korea). The other reference standard was purchased from Korea Food and Drug Administration. The purity (%) of five reference standards had been above 98%. HPLC grade solution, methanol, water, and regents were purchased from from J. T. Baker (USA). All of the plant materials were purchased from Kyungdong traditional herbal market (Seoul). Four commercial Bangpungtongsung-san samples were obtained from four different herbal medicine companies.

### Chromatography condition

Analysis was performed on the HPLC system (Dionex, Germany). The HPLC system was equipped with a pump (LPG 3X00), auto-sampler (ACC-3000), column oven, and diode-array UV/VIS detector (DAD-3000(RS)). The output signal of the detector was recorded using a Dionex Chromelon ^TM^ Chromatography Data System. The separation was executed on a Dionex C _18_ column (5 μm, 120 µ, 4.6 mm × 150 mm). The mobile phase was composed of water and methanol with the gradient elution system at a flow rate of 1.0 ml/min. The injection volume was 20 μl. The detection UV wavelength was set at 230, 240, 254, and 280 nm. The column temperature was set at 25°C.

### Preparation of standard solutions and sample

The standard stock solutions of five marker components, paeoniflorin, 6-gingerol, decursin, geniposide, and glycyrrhizin, were prepared by dissolving at the concentration of 100 μg/ml (paeoniflorin), 100 μg/ml (6-gingerol), 100 μg/ml (decursin), 250 μg/ml (geniposide), and 250 μg/ml (glycyrrhizin) in 10 ml of 60% methanol. Working standard solutions were made by diluting the standard stock solution with methanol. These working solutions were mixed and applied to calibration curves. The standard stock solutions and working solutions were stored at 4°C. The Bangpungtongsung-san sample consisted of 0.40 g of *Angelica gigas*, 0.40 g of *Paeonia lactiflora*, 0.40 g of *Cnidium officinale*, 0.40 g of *Gardenia jasminoides*, 0.40 g of *Forsythia viridissima*, 0.40 g of *Mentha arvensis*, 0.40 g of Zingiber officinale, 0.40 g of Schizonepeta tenuifolia, 0.40 g of Saposhnikovia divaricata, 0.40 g of Ephedra sinica, 0.50 g of Rheum undulatum, 0.50 g of *Natrii sulfas*, 0.67 g of *Atractylodes japonica*, 0.67 g of *Playtcodon grandiflorum*, 0.67 g of *Scutellaria baicalensis*, 0.67 g of *Glycyrrhiza uralensis*, 1.00 g of *Gypsum*, and 1.07 g of talcum. These herbs were extracted by a heating extraction method (in water 8-10 times the weight of herbs) for 3-4 h in 90-100°C. The extract was evaporated under vacuum and was prepared in the form of powder by a freeze-dryer. A total of 20 mg of the prepared Bangpungtongsung-san sample and four commercial Bangpungtongsung-san samples were weighed precisely and were dissolved in 60% methanol at a concentration of 2 mg/ml. Before HPLC analysis, the sample preparation was filtered through a 0.45-μm filter.

## RESULTS AND DISCUSSION

### Optimization of suitable HPLC-DAD conditions

**Figure 2:**
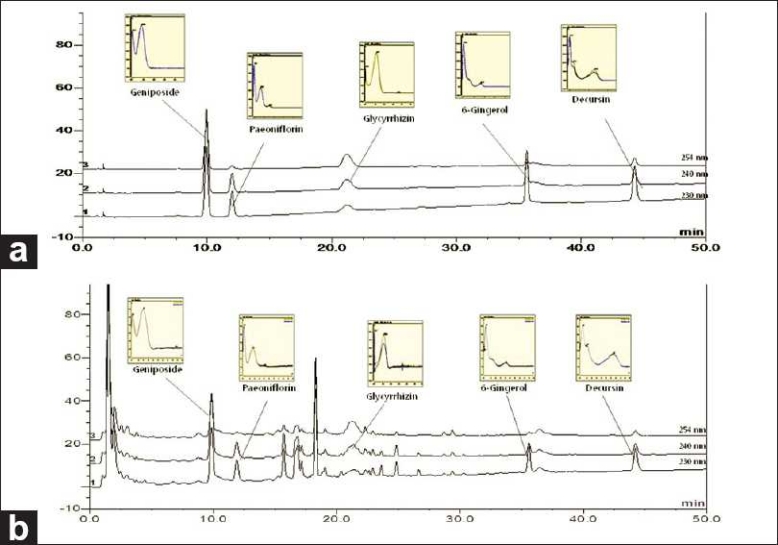
The HPLC chromatogram of the standard mixture (a) and Bangpuntongsung-san sample (b)

To precisely and exactly analyze five marker components in Banhpungtongsung-san, a suitable HPLC system condition, such as chromatographic column, mobile phase elution system, UV wavelength of detector, was established. In general, a reverse phase column was applied to assay chemical components in natural products. We selected C _18_ as one. The mobile phase consisting of methanol (A) and water (B) was tested in various gradient systems and an adequate gradient ratio was selected. The gradient volume of solvent B was 75-70% at 0-10 min, 70-50% at 10-20 min, 50-40% at 20-30 min and 40-30% at 30-50 min. The UV wavelength of the DAD detector was tested at 230, 240, 254, and 280 nm. A wavelength of each component selected most of the absorbed UV wavelength in the UV spectrum. Paeoniflorin, 6-gingerol, and decursin were 230 nm, geniposide was 240 nm, and glycyrrhizin was 254 nm. The peak of each compound was confirmed by comparing the retention time and UV spectrum of each marker constituent. Retention times for peaks of geniposide, paeoniflorin, glycyrrhizin, 6-gingerol, and decursin were 10.03, 12.10, 20.83, 35.81, and 44.51 min, respectively [Figure 2a]. The Bangpungtongsung-san sample was analyzed by this HPLC method and a chromatogram of sample showed that five marker components were separated effectively [Figure 2b].

## METHOD VALIDATION

### Calibration curves, limit of detection, and limit of quantification

Method validation was executed by linearity, precision and accuracy test on the basis of ICH guidelines. Linearity was confirmed by the correlation coefficient (R^2^). To calculate the regression equation, five different concentrations of the standard solution were used to draw up a calibration curve. The regression equation formed was Y = ax + b (a - slope of the calibration curve, b - intercept of the calibration curve); Y axis was the value of peak area and x axis was the concentration of marker components. The correlation coefficient of each marker component’s calibration curve showed good linearity (R^2^ > 0.9959). According to the calibration curve, the limit of detection (LOD), and the limit of quantification (LOQ) were determined as LOD = 3.3 × (SD/Slope) and LOQ = 10 × (SD/Slope), where SD is the standard deviation of the response and slope is slope of the calibration curve. The values of LOD and LOQ were within a range of 0.01-0.17 and 0.04-0.53 μg/ml, respectively [Table 1].

**Table 1:** The linearity, correlation coefficient (R^2^), limit of detection and limit of quantification of the compounds studied

Components	Linear range (µg/ml)	Regression Equationa	R^2^ (n=5)	LOD (µg/ml)	LOQ (µg/ml)
Paeoniflorin	0.2 - 20	Y = 0.5714 *x* + 0.1290	0.9959	0.01	0.04
6-Gingerol	0.2 - 20	*Y* = 0.2377 *x* + 0.0314	0.9976	0.06	0.19
Decursin	0.2 - 20	*Y* = 0.4249 *x* – 0.0450	0.9994	0.10	0.30
Geniposide	0.5 - 50	*Y* = 0.4643 *x* + 0.2668	0.9985	0.03	0.08
Glycyrrhizin	0.5 - 50	*Y* = 0.1920 *x* + 0.0489	0.9983	0.17	0.53

^a^Y: peak area, *x*:concentration (µg/ml), LOD: limit of detection, LOQ: limit of quantification

### Precision and accuracy

To measure precision and repeatability, the intra- and interday test was executed by analyzing three different concentrations of five standard solutions. The test was carried out five times in 1 day and in an interval of 1 day during 5 days (1, 3, 5 days), respectively. Precision and repeatability were confirmed by the relative standard deviation (RSD) value of intra- and interday. The RSD was calculated by standard deviation over the measured amount multiply by 100. The RSD values of intra- and interday were 0.03-0.43% and 0.04-1.26%, respectively [Table 2]. To evaluate accuracy, a recovery test was carried out. Three different concentrations of five marker components were added in 20 mg/ml of the Bangpungtongsung-san sample and analyzed three times. Each recovery value ranged from 94.46% to 107.70% and RSD values varied between 0.03% and 2.21% [Table 3].

**Table 2:** Analytical results of intra- and inter-day test

Components	Concentration (µg/ml)	Intra-day (n=5)		Inter-day (n=5)	
		Mean ± SD (µg/ml)	RSD (%)	Mean ± SD (µg/ml)	RSD (%)
Paeoniflorin	6.00	6.85 ± 0.017	0.43	6.58 ± 0.003	0.04
	2.40	2.58 ± 0.001	0.06	2.58 ± 0.005	0.21
	1.20	1.19 ± 0.002	0.21	1.18 ± 0.007	0.61
6-Gingerol	22.00	22.86 ± 0.010	0.18	22.24 ± 0.021	0.10
	8.00	7.03 ± 0.005	0.07	6.99 ± 0.028	0.41
	4.00	5.36 ± 0.008	0.15	5.38 ± 0.024	0.44
Decursin	12.00	12.89 ± 0.010	0.018	12.79 ± 0.011	0.08
	10.00	10.90 ± 0.007	0.07	10.86 ± 0.023	0.22
	5.00	5.16 ± 0.005	0.10	5.13 ± 0.021	0.42
Geniposide	25.00	26.41 ± 0.009	0.07	26.02 ± 0.011	0.04
	10.00	11.44 ± 0.004	0.03	10.99 ± 0.013	0.12
	5.00	5.04 ± 0.006	0.13	5.10 ± 0.014	0.27
Glycyrrhizin	25.00	23.73 ± 0.010	0.22	26.44 ± 0.040	0.15
	10.00	9.21 ± 0.003	0.03	10.08 ± 0.026	0.26
	5.00	6.68 ± 0.003	0.05	6.71 ± 0.085	1.26

**Table 3:** Analytical results of accuracy test

Components	Spiked Concentration (µg/ml)	Measured Concentration (µg/ml)	RSD (%)	Recovery^a^ (%)
Paeoniflorin	3.00	3.18 ± 0.02	0.71	105.84
	1.20	1.25 ± 0.01	0.65	104.32
	0.60	0.60 ± 0.01	2.21	100.47
6-Gingerol	14.00	13.95 ± 0.03	0.24	97.10
	2.50	2.45 ± 0.02	0.89	98.11
	1.25	1.30 ± 0.01	1.09	103.71
Decursin	6.00	6.46 ± 0.03	0.39	107.70
	5.00	5.25 ± 0.03	0.49	105.10
	2.50	2.61 ± 0.02	0.86	104.33
Geniposide	12.50	13.14 ± 0.01	0.03	105.10
	5.00	5.04 ± 0.01	0.18	100.72
	2.50	2.43 ± 0.02	0.83	97.40
Glycyrrhizin	12.50	13.21 ± 0.04	0.32	105.70
	5.00	4.87 ± 0.03	0.55	94.46
	2.50	2.38 ± 0.01	0.51	95.27

a Recovery (%) = (concentration found – original concentration)/concentration spiked ×100 %

### Analysis of the Bangpungtongsung-san sample

**Figure 3:**
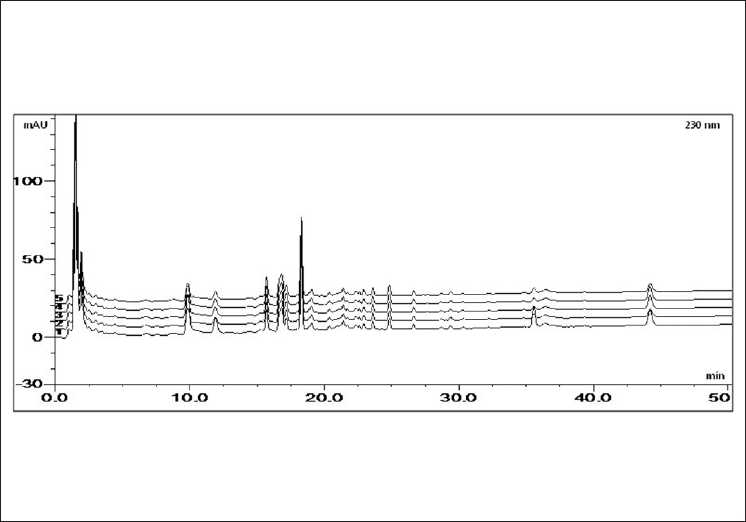
The HPLC chromatogram of the Bangpungtongsung-san sample (1: BPTSS – A, 2: BPTSS – B, 3: BPTSS – C, 4: BPTSS – D, 5: BPTSS – E)

The developed HPLC method was applied to the prepared Bangpungtongsung-san sample and four samples were obtained from different herbal medicine companies [Figure 3]. The contents of five marker components were calculated from the calibration curve of the standards. The results indicated that differences appeared in the amounts of marker components in Bangpungtongsung-san samples A-E. The content of marker components in herbs might have been affected by many factors such as collection time or cultivation environment and the manufacturing process of the prescription such as Bangpungtongsung-san [Table 4].

**Table 4:** Components of five marker compounds in commercial Bangpungtongsung-san samples

	Content (mg/g)				
	Paeoniflorin	6-Gingerol	Decursin	Geniposide	Glycyrrhizin
BPTSS-A	1.30 ± 0.08	0.07 ± 0.02	0.94 ± 0.06	6.84 ± 0.41	4.66 ± 1.52
BPTSS-B	1.05 ± 0.02	0.03 ± 0.08	1.18 ± 0.21	6.26 ± 0.02	4.20 ± 0.11
BPTSS-C	1.27 ± 0.11	0.01 ± 0.04	1.27 ± 0.13	6.05 ± 0.03	4.56 ± 0.22
BPTSS-D	1.25 ± 0.05	0.05 ± 0.04	0.80 ± 0.09	6.13 ± 0.06	4.60 ± 0.13
BPTSS-E	1.19 ± 0.12	0.05 ± 0.09	0.93 ± 0.07	6.51 ± 0.06	4.45 ± 0.15

BPTSS-A: Prepared BPTSS sample, BPTSS-B~E: Four commercial BPTSS sample

## Conclusions

In this paper, the simultaneous determination of five markers, paeoniflorin, 6-gingerol, decursin, geniposide, and glycyrrhizin, in Bangpungtongsung-san was established by HPLC/DAD. The validation of the developed method verified its reliability and stability. The developed method successively separated off five marker components in Bangpungtongsung-san samples. In conclusion, this HPLC/DAD method was useful for the quality control of Bangpungtongsung-san.
